# A new framework on climate-induced food-security risk for small-scale fishing communities in Tanzania

**DOI:** 10.1007/s12571-024-01472-x

**Published:** 2024-07-19

**Authors:** Lara Paige Brodie, Smit Vasquez Caballero, Elena Ojea, Sarah F. W. Taylor, Michael Roberts, Patrick Vianello, Narriman Jiddawi, Shankar Aswani, Juan Bueno

**Affiliations:** 1 Centro de Investigación Mariña (CIM), Future Oceans Lab, Universidade de Vigo, Campus Lagoas Marcosende, 36310 Vigo, Spain; 2 Center for Applied Economics and Strategy, RTI International, Research Triangle Park, Durham, NC 27709 USA; 3 National Oceanography Centre, Southampton, SO14 3ZH UK; 4 Nelson Mandela University, Port Elizabeth, South Africa; 5 Institute of Marine Sciences, UDSM, Zanzibar, Tanzania; 6 Rhodes University, Grahamstown, South Africa

**Keywords:** Food security, Climate change, Small-scale fisheries, Risk assessment, Western Indian Ocean

## Abstract

Food insecurity is a pressing issue facing our world, particularly affecting coastal communities who rely on marine resources. The problem is further compounded by the rapidly changing climate, a deteriorating environment and growing human populations. It is essential to evaluate this issue accurately to reduce risk and improve the situation of coastal communities, especially in countries with less socioeconomic development. To this end, we develop a food security social-ecological risk assessment framework for developing communities in coastal areas of the Western Indian Ocean facing a changing environment. The framework integrates local ecological knowledge, expert scientific opinion, survey data, and satellite sea surface temperature (SST) and chlorophyll-a observation. We conducted a local-scale case study in four regions in Tanzania; Mafia, Pemba, Tanga, and Unguja, revealing that they face moderate to high risk levels of food insecurity. The highest risk was observed in the island communities of Pemba and Unguja, while the communities of Mafia and Tanga had the lowest risk due to lower exposure and sensitivity to climate change. Our results show that recognizing the key differences across risk components is crucial in identifying effective intervention strategies for local practitioners. This study highlights the need for detailed assessments to provide accurate information on local-scale food security dynamics, specifically when assessing impacts induced by environmental and climatic changes.

**Supplementary Information:**

The online version contains supplementary material available at 10.1007/s12571-024-01472-x.

## Introduction

Ocean sustainability is critical for the survival of humanity (Brodie Rudolph et al., 2020). However, marine ecosystems are facing unprecedented cumulative pressures from human activities and anthropogenic climate change (Jouffray et al., 2020; Tigchelaar et al., 2021). To achieve sustainability through marine management, it is crucial to recognize that the ocean is a social-ecological system, in which people and nature are linked and interdependent (Fischer et al., 2015; Salgueiro-Otero & Ojea, 2020). Climate change is having significant effects on the global ocean, including rising ocean temperatures, ocean acidification, and sea level rise among other impacts (Cooley et al., 2022; Sumaila & Tai, 2020). These changes will have worldwide effects on the marine environment, including target species important for small-scale fisheries (SSF), thereby impacting food security of local fishing communities (Sekadende et al., 2020; Taylor et al., 2019).

One of the major consequences of these changes is the threat to food security worldwide (Campbell et al., 2016; Costello et al., 2020). SSFs play a crucial role in supporting the livelihoods of more than half a billion people and up to three billion people rely on fish as a major component of their diet (FAO, 2010). As fish are a rich source of protein and micronutrients, they are important not only as a source of food but also as a source of nutrition (Hicks et al., 2019). The nutritional content of fish is increasingly being recognized as a key element to address global nutritional deficiencies, which lead to child underdevelopment, growth stunting, and increased mortality rates, especially in low-income developing countries (Golden et al., 2016; Hicks et al., 2019; Willett et al., 2019). With the predicted worsening of food and nutrition insecurity problems because of climate change (Costello et al., 2020; Schmidhuber & Tubiello, 2007), it is of utmost importance to quantify, document and respond to the effects of climate change on food security, particularly in coastal developing communities where there is a high dependency on marine resources (Teh & Sumaila, 2013). Thus, food and nutrition policies need to consider the contributions of fisheries to the well-being of the most vulnerable in society, such as resource dependent communities (Hicks et al., 2019).

Previous research has shown that risk assessments are useful tools for understanding the specific weaknesses of social-ecological systems (Reisinger et al., 2020). Risk assessments are indicator-based approaches that integrate both social and ecological information which enable ranking different units of analysis (species, habitats, social groups, etc.) according to the risk of suffering the impact of a given stressor (Reisinger et al., 2020). The results of risk assessments can be used in policy development and relevant adaptation planning (Allison et al., 2009; IPCC, 2013; Marshall et al., 2010). However, there is a gap in the literature regarding local-scale food security vulnerability/risk assessments specifically focusing on low-income coastal communities. Previous food security assessment studies have been conducted at the national scale (Ding et al., 2017; Hughes et al., 2012). These national-scale assessments fail to document local-scale food security dynamics and are not able to identify specific sensitivities and adaptive capacities of local communities (Cinner et al., 2012; McClanahan et al., 2015). In addition, aggregated statistics can mask the severity of the food crisis facing communities and fail to reveal important differences between communities within the same country (Taylor et al., 2019). Therefore, there is a need for granular and detailed assessments that can provide accurate information on local-scale food security dynamics in low-income coastal communities.

The impact of climate change, among other environmentally degrading processes, on food security is a pressing issue that requires attention, particularly in developing coastal communities that rely heavily on marine resources for food and livelihoods. The Western Indian Ocean (WIO) is a prime example of such a region, where between 30 to 60 million people are susceptible to rapid climate induced changes (Sekadende et al., 2020; UNEP-Nairobi Convention & WIOMSA, 2015). Tanzania, a least developed country within the WIO region, is home to many coastal communities that rely on artisanal and subsistence fishing practices for their sustenance (IPCC, 2013; March & Failler, 2022; Taylor et al., 2019). The WIO is experiencing some of the fastest climate-induced changes globally, including significant warming and an increase in intensity and duration of extreme marine heatwave events (Sekadende et al., 2020). Changes to the monsoon seasons are also projected, which will impact the ecosystem on which coastal communities depend (Mahongo & Shaghude, 2014; Schott et al., 2009). These changes are expected to greatly impact food availability, access, utilization, and stability in the region (Huang et al., 2017; IPCC, 2013; Jacobs et al., 2021; Roxy et al., 2014, 2015). Thus, performing a food security risk assessment is both timely and relevant to inform policy and adaptation efforts. The overall purpose of this paper is to conduct a detailed assessment of the food security vulnerabilities and risks faced by low-income fishing communities in Tanzania, and to identify specific sensitivities and adaptive capacities of these communities in response to the impacts of climate change. Results can be used to demonstrate the necessity of a more granular assessment of impacts to inform appropriate adaptive policy and community responses.

To achieve our goal, this paper pursues three main objectives that are aimed at assessing and addressing the climate-induced food security risk (CFSR) in fishing communities in Tanzania. The first objective was to develop a framework that could measure the impact of climate change on the availability and access to sufficient food. This was achieved by adopting and tailoring an existing social-ecological vulnerability assessment framework (Aswani et al., 2018; Cinner et al., 2012). The second objective was to operationalize this framework by integrating multiple data sources to measure the dimensions of risk (hazard, exposure, sensitivity, and adaptive capacity) of the fishing communities to climate risk. The third objective was to provide guidance for local adaptation and to inform policy and management based on the results. Cumulatively, this paper contributes to the risk assessment literature by providing a framework to assess the impacts of climate in different resource extraction environments. In addition, the paper contributes to the food security literature by providing a case study showing the nuanced vulnerability of communities dependent on marine resources, highlighting the importance of both local knowledge and specificity in identifying climate risks and providing policy recommendations unique to the local context.

## Methods and materials

### Study region

**Fig. 1:**
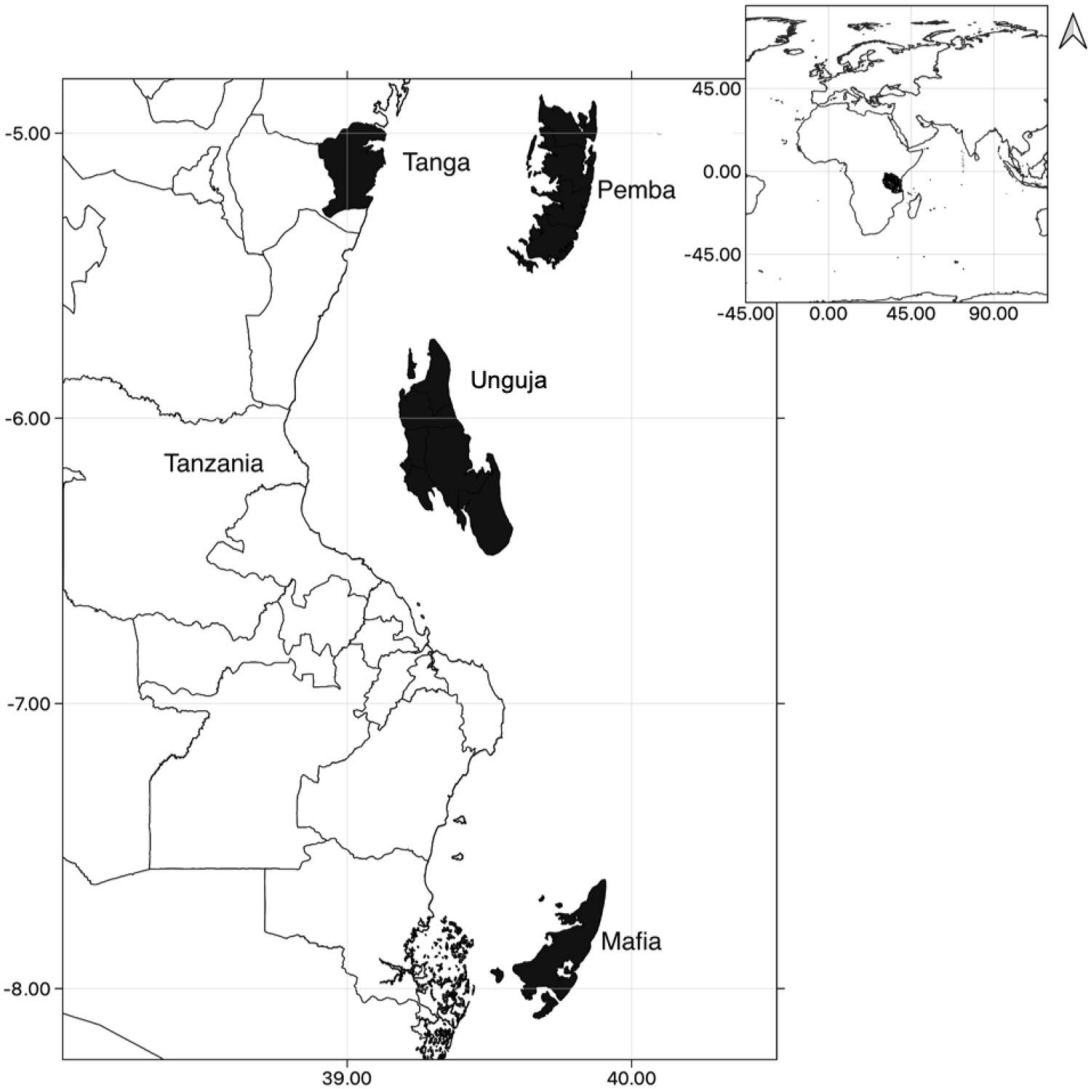
Study region. The four study sites of Mafia, Pemba, Tanga and Unguja are highlighted in dark

The study focuses on four coastal regions in Tanzania: Tanga, Mafia, Pemba, and Unguja, with the first located on the mainland and the others three are islands (see Fig. 1). All four regions consist of multiple villages within each, and here after the four regions are referred to “communities”. These communities offer a relevant case study because they rely heavily on food from the sea, and the impacts of climate change on their food security and livelihoods have been well-documented (IPCC, 2013; Sekadende et al., 2020; Taylor et al., 2019). Furthermore, these communities present significant heterogeneity in terms of food security (Taylor et al., 2021). Tanga is surrounded by areas of coral reefs, seagrass beds, mangroves, and depth changes, and has been heavily affected by dynamite fishing (Samoilys & Kanyange, 2008). Unguja and Pemba are surrounded by abundant coral reefs with large drop-offs to 40 m. Unguja is a well-known tourist destination (Benansio & Jiddawi, 2016), with a growing tourism industry which accounts for 25% of Tanzania’s GDP (Lange, 2015). Meanwhile, Mafia is geographically isolated, and the eastern coastline has a steep continental slope under the influence of the East African Coastal current thus limiting their access to marine resources (Obura, 2005). Limited alternative livelihoods are a common characteristic of these communities.

### The climate-induced food security risk (CFSR) framework

**Fig. 2:**
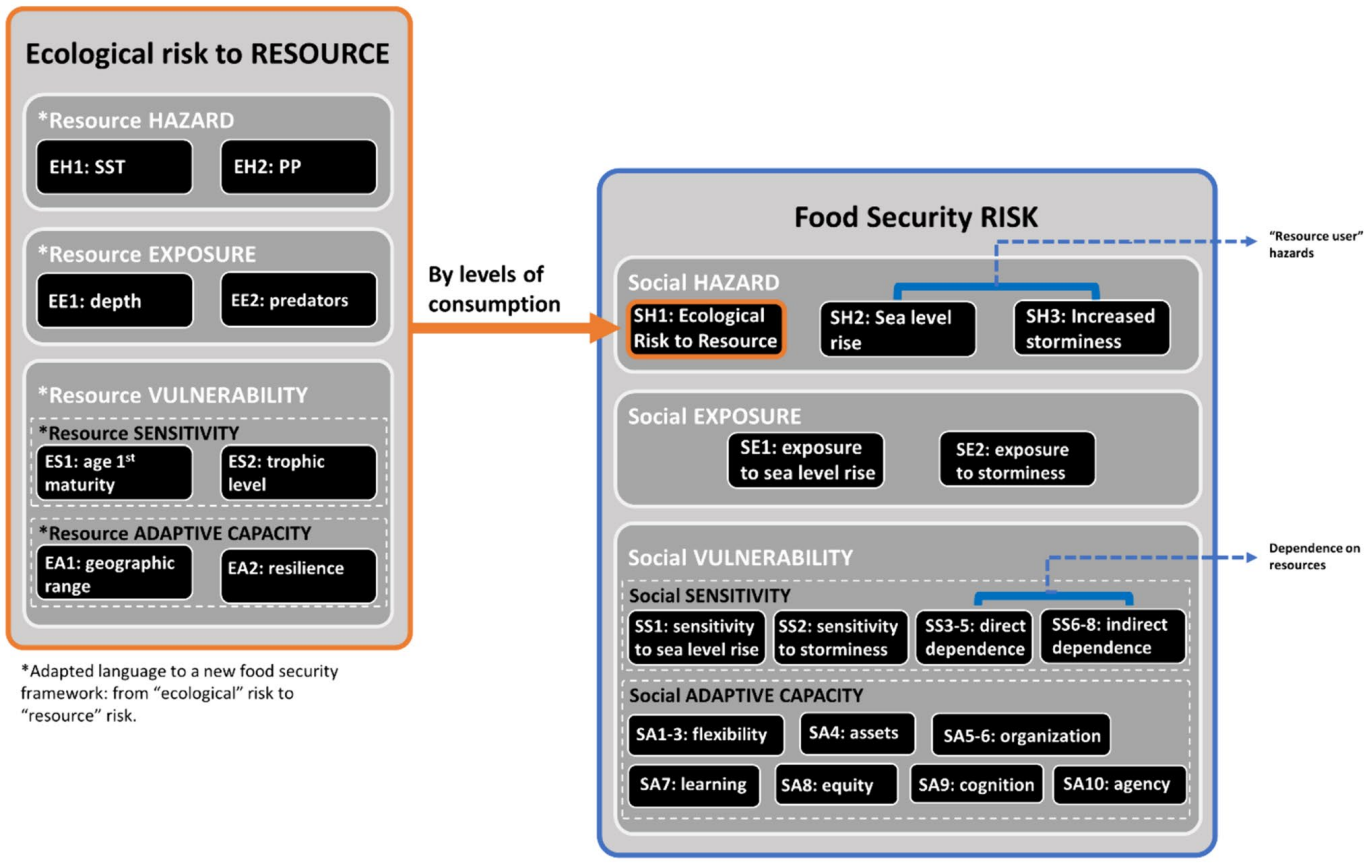
Climate-Induced Food Security Risk Framework (CFSR). The figure illustrates the conceptual framework that was developed and utilized to evaluate the climate-induced food security risk in four coastal communities in Tanzania. The left-hand figure displays the total ecological risk to the resources, which is nested within the overall final CFSR framework on the right-hand side. This ecological risk resource serves as one indicator for “hazard.” To calculate the ecological risk resource, four dimensions are considered: resource hazards (EH1 “Sea surface temperature (SST)” & EH2 “Primary Production – PP”), resource exposure (EE1 & EE2), resource sensitivity (ES1 & ES 2), and resource adaptive capacity (EA1 & EA2). Four dimensions are used to describe the overall CFSR: social hazard (SH1, SH2, SH3), social exposure (SE1 & SE2), social sensitivity (SS1 – SS8). The darker rectangles in the figure enumerate each indicator for every dimension

We have adopted and tailored the climate risk conceptual framework proposed by the Intergovernmental Panel on Climate Change in 2014 (IPCC, 2014) to investigate food security in SSF communities in the study region. Specifically, we embedded the IPCC approach into the social-ecological vulnerability assessment framework of Cinner et al., (2012) and applied it to local scale assessments with a specific focus on food security. Our framework encompasses the three dimensions of risk, as outlined by the IPCC (2014): hazard, exposure, and vulnerability, where vulnerability is defined as a function of sensitivity and adaptive capacity, as illustrated in Fig. 2. For the purpose of building a food security risk framework, in this study, hazard refers to physical events caused by natural or human-induced factors that adversely affect resources and resource users and consequently fishers’ ability to catch, consume, and sell fish. Climate hazards are known to have both direct and indirect impacts on food security, affecting not only the availability of food (Allison et al., 2009), but also the access to crucial food resources (Cinner et al., 2013). Exposure is broadly defined as the presence of fishing communities, fish species, and economic, social, or cultural assets in areas and situations that may be negatively impacted by climate hazards (IPCC, 2014). Sensitivity measures resilience to climate hazards and social dependence on fish (Ding et al., 2017), considering both direct effects through food consumption and indirect effects through income generated from fish sales. Adaptive capacity, on the other hand, assesses the ability of fishing communities to adapt to the changing abundance and distribution of key resources they depend on. Finally, vulnerability encompasses sensitivity and adaptive capacity and assesses the weaknesses of the fisheries system and its ability (or lack thereof) to manage food security impacts (Sharma & Ravindranath, 2019).

We used the three dimensions of risk to calculate the climate-induced food security risk (CFSR) index for each of the four communities using the following formula:

1 $${CFSR}_{i}=mean \left({SH}_{i}+ {SE}_{i}\right)+\left({SS}_{i}-{SAC}_{i}\right),$$

where *i* denotes an individual fishing community and *SH*, *SE*, *SAC*, and *SS* denote social hazard, social exposure, social adaptive capacity, and social sensitivity, respectively.

### Data sources

To assess the climate risk index for food security in all communities, we employed a combination of information sources, including a survey, publicly available databases, and expert elicitation. In 2018, we administered a local-scale survey as part of the Global Challenges Research Fund SOLSTICE-WIO project (Taylor et al., 2021). The survey comprised more than 250 questions and garnered responses from 293 male fishers across the four study sites (Mafia, Pemba, Tanga, and Unguja), with 90, 49, 52, and 102 responses, respectively. The surveyed fishers ranged in age from 18 to 78, with an average age of 32 to 38 across all communities. The survey included a household questionnaire that incorporated open-ended, semi-structured, and structured questions related to economic dependency, fishing practices, and perceptions of community-level vulnerabilities. We utilized publicly available datasets, including the Sea Surface Temperature (SST) and data on Chlorophyll-a (chl-a) from The GlobColour Project.^1^ Both physical climate datasets were collected for the period of 2001 to 2021 and were converted to monthly time series. Finally, in March 2021, we consulted marine ecology experts to obtain data through expert elicitation to fill gaps in biological data from Fishbase^2^ related to species life-history characteristics.

### Operationalization of the CFSR

To implement the CFSR framework, we constructed a set of indicators to evaluate each of the three dimensions of risk as demonstrated in Eq. 1. In the next subsection, we will provide a comprehensive explanation of the quantification process for each dimension.

#### Quantifying social hazard

The social hazard risk dimension is measured using three different indicators, namely ecological risk to resource, sea-level rise, and storms (indicated as SH1, SH2, and SH3 in Table 1). Ecological risk to resource measures resource hazards, while storms and sea-level rise measure resource user hazards. The ecological risk to resource (*ERR*) indicator is calculated using the following formula:

2 $${ERR}_{i}=mean \left({RH}_{i}+{RE}_{i}\right)+\left({RS}_{i}-{RAC}_{i}\right),$$

where *i* denotes a fishing community and *RH*, *RE*, *RS*, and *RAC* refer to consumed resource hazard, resource exposure, resource sensitivity, and resource adaptive capacity, respectively.

**Table 1:** List of dimensions, indicator, sub indicator, and data sources. Summary of the indicators, sub-indicators, survey questions, and other data sources used to measure the social hazard, exposure, sensitivity, and adaptive capacity of four developing coastal communities in Tanzania. The codes are used to describe each indicator of each dimension

Dimension	Indicator	Code	Sub-indicator	Data source	Question/Data	Unit of measurement
SOCIAL HAZARD	Ecological risk to resource	SH1	-	Multiple	-	Combination
	Seal level rise	SH2	-	Survey	Has the shoreline in your village/area changed over the years?	Yes/No
	Increasing storminess	SH3	-	Survey	Has there been a storm/cyclone in the last 5 years in your area?	Yes/No
SOCIAL EXPOSURE	Exposure to sea level rise	SE1	-	Survey	Have you noticed places in your area where the shoreline has been eroded by the sea?	Yes/No
	Exposure to increasing storminess	SE2	-	Survey	Were you directly impacted by the cyclone/large storm?	Yes/No
SOCIAL SENSITIVITY	Sensitivity to Sea Level Rise	SS1	-	Survey	Have you noticed changes to your livelihood as a result of the shoreline erosion?	Yes/No
	Sensitivity to increasing storminess	SS2	-	Survey	How bad was the cyclone/large storm damage to your household?	Scale 1–4
			-	Survey	How many fishing days did you lose to the large storm/cyclone in the last year?	Scale 1–4
	Direct dependence	SS3	Dependence equation	Survey	Days per week consuming fish/ Days per week consuming meat and fish	Percentage
		SS4	Nutrition indicator	External + Survey	Nutrition level of the functional seafood groups most eaten by each household	Values
		SS5	Personal perception	Survey	How possible would it be to feed your family if you did not fish?	Scale 1–4
	Indirect dependence	SS6	Income dependence	Survey	How much of your household dependence is derived from fishing?	Percentage
		SS7	Employment dependence	Survey	How important is fishing as an economic activity in your community?	Scale 1–4
		SS8	Wealth dependence	Survey	How likely is it that you would have to sell your house if fishing fails?	Scale 1–4
SOCIAL ADAPTIVE CAPACITY	Flexibility	SA1	Personal	Tanzania Govt Statistics	Life expectancy at birth values	Values
				Survey	How likely are you to adapt to changes in comparison to others you know?	Scale 1–4
		SA2	Occupational	Survey	Have you changed jobs in the last 5 years?	Yes/No
				Survey	How many options for different types of work do you have?	Scale 1–4
				Survey	How useful would your skills be in setting up a business other than fishing?	Scale 1–4
		SA3	Institutional	Survey	How many markets are there to buy fish?	Value
				Survey	How many markets are there to sell fish?	Value
	Assets	SA4	Asset Wealth Index	Survey	Combination of many questions	Combination
	Social Organization	SA5	Community organization	Survey	Does your community have a marine resource management system?	Yes/No
				Survey	How well enforced are the fishing rules in the area?	Scale 1–4
		SA6	Government relations	Survey	How well linked is the community to government departments or academic institutions to receive information about fishing?	Scale 1–4
				Survey	Are there institutions or government departments working on climate change or facilitating adaptation?	Yes/No
	Learning	SA7	Level of education	Survey	Highest education level you have achieved?	Category
			Recognition of causal agents impacting marine resources	Survey	Have you heard of climate change?	Yes/No
				Survey	Have you noticed changes to your communities over your lifetime?	Yes/No
			Local ecological knowledge	Survey	How much do you know about the environment in which you live?	Scale 1–4
				Survey	How important is it to pass LEK to future generations?	Scale 1–4
	Equity	SA8	Gender equity	Survey	Are women in leadership roles in the community?	Yes/No
				Survey	How equal is women’s access and control over their livelihoods and resources compared to men?	Scale 1–4
	Social cognition	SA9	Risk attitudes	Survey	How safe do you feel you are in your main occupation in the context of climatic exposure?	Scale 1–4
				Survey	How much of a risk do you think the environment poses to the community you live in, in terms of livelihood and income?	Scale 1–4
				Survey	How confident are you that things will turn out regardless of the challenges and changes you confront?	Scale 1–4
	Agency	SA10	Independence	Survey	Would you say being independent is the worst or best thing about being a fisher?	Scale 1–4
				Survey	How often are you thinking of new and better ways to improve your fishing business/livelihood?	Scale 1–4
				Survey	How possible is it for you to make a personal difference in improving the health of the marine environment in this area?	Scale 1–4

**Fig. 3:**
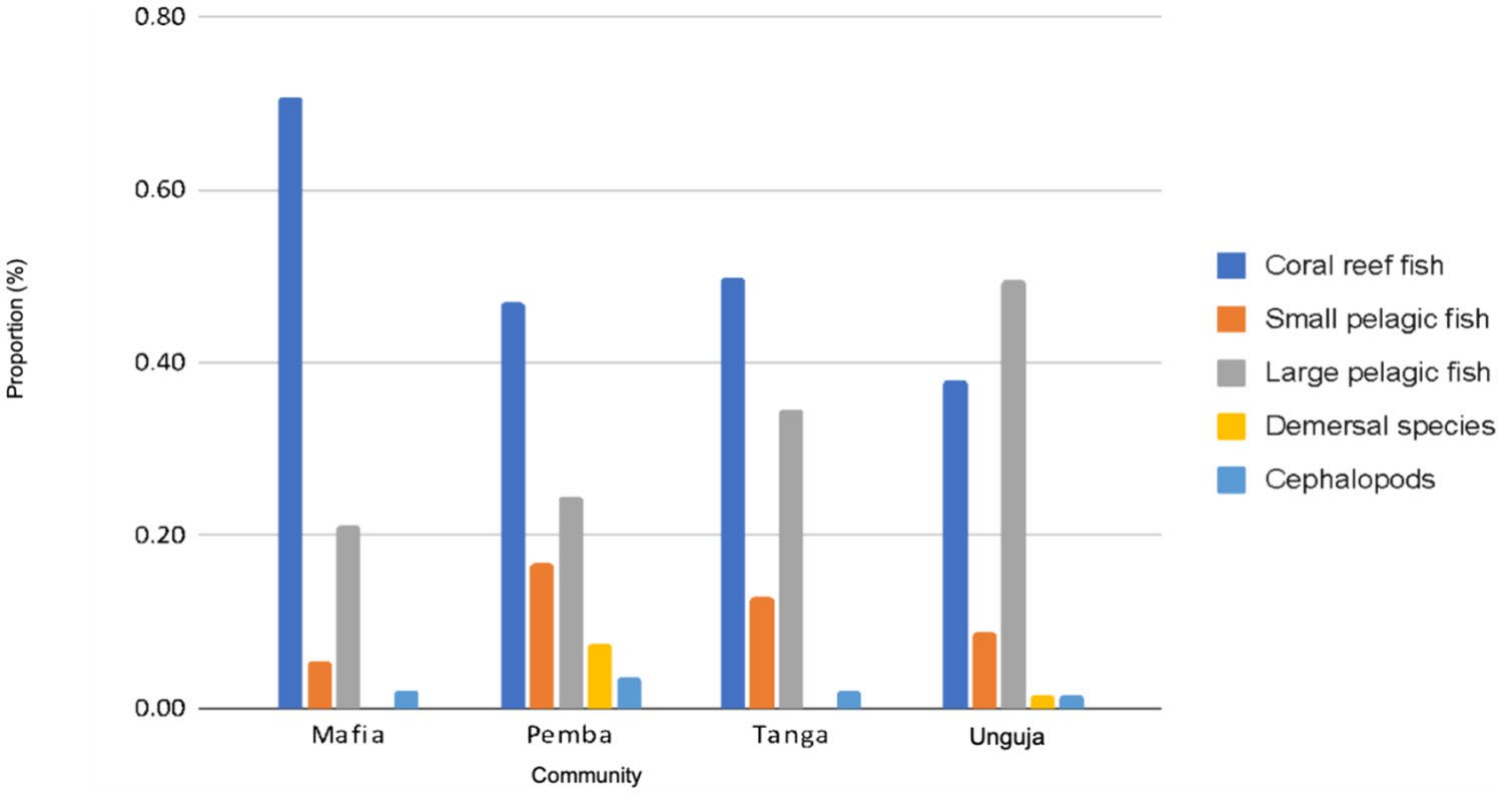
The proportion of fish per functional seafood group consumed by communities

To identify the consumed resources of each community, we obtained the primary fish products consumed by households and their respective communities from the survey data. These were then classified into five functional seafood groups, namely: coral reef fish, small pelagic fish, large pelagic fish, demersal species, and cephalopods. This classification was necessary due to the lack of information of some species and shared life histories by species from the same functional seafood group (Gaichas et al., 2014). Next, the proportions of each functional seafood group consumed by each community were calculated based on the frequency with which a functional seafood group was reported as the most commonly consumed, the results are presented in Fig. 3. Table S1 in the Supplementary Information (SI) shows the classification of fish families by functional seafood group.^3^

To quantify the hazard scores of the consumed resources (resource hazard), we identified two critical climatic hazards: temperature, which was measured as the rise in sea surface temperature (SST), and changes in ocean productivity, which were measured as alterations in primary productivity (Jacobs et al., 2021; Jebri et al., 2020; Sekadende et al., 2020). Since both variables have their respective units of measurement, we calculated an index of variability (IV) using Bueno-Pardo et al. (2021) formula:

3 $$IV=\frac{\mu FUT- \mu REF}{\sigma REF}$$

where *μREF* and *σREF* denote the variables’ average and its standard deviation from the reference period of 2001 to 2011. *μFUT* represents the variables’ average between 2011 and 2021. The index of variability is devised to assess the anticipated extent of variation in hazards relative to the reference period while adjusting for the natural variability of the hazard during the 2001 to 2011 period. Since the inter-community distances are relatively short (approximately 50 km), a singular value for each hazard was computed across all communities.

To assess the resource exposure, sensitivity, and adaptive capacity of the five functional seafood groups to climate hazards we use a combination of relevant literature and expert opinions (Table 2). To quantify the exposure of the five functional seafood groups to the two identified climate hazards, we selected indicators related to the life-history traits, as found in the literature (Bueno-Pardo et al., 2021; Cinner et al., 2013). Each indicator was assessed using a three-level categorical scale (low, intermediate, high); Table 2 summarizes the evaluation criteria. We assigned categorical values to indicators based on findings from the literature or according to three experts on tropical fish ecology. Additionally, as the assessment is conducted at the functional seafood group level, the criteria for evaluation were obtained from a combination of the values of the main families represented within each group (refer to tables 7 and 8 in the SI). The resources sensitivity and adaptive capacity of functional seafood groups were evaluated using two indicators each, as detailed in Table 2. Age at maturity and trophic level were utilized to estimate resource sensitivity as done in previous literature (Bueno-Pardo et al., 2021). Additionally, extension of occurrence range and resilience to fisheries were used to assess adaptive capacity. Data for both adaptive capacity indicators was obtained from Fishbase^2^. “Resilience to fisheries” was calculated using the inverted “vulnerability to fisheries” value (Cheung et al., 2005). In accordance with exposure indicators, existing literature was referenced to evaluate the levels of these indicators (i.e., low, medium, high) as well as expert opinions (Bueno-Pardo et al., 2021; Hare et al., 2016; Pecl et al., 2014; Wang et al., 2020). Table S3 in the SI lists all the values of the estimated resource exposure, sensitivity, and adaptive capacity of each functional seafood group.

**Table 2:** Indicators and the criteria used to determine the overall ecological risk to resource

Component	Code	Indicator	Risk level		
			**LOW**	**MODERATE**	**HIGH**
Resource Exposure	EE1	**Sea Surface Temperature**	Demersal species occurring mainly below 50 m depth	Demersal species occurring mainly above 50 m depth	Coral-reef-associated species or species with pelagic adults, larvae, and eggs
	EE2	**Primary Productivity**	Higher level predators	Species feeding on macroscopic invertebrates, fish larvae, algae grazers, etc	Filter-feeding species
Resource Sensitivity	ES1	**Age at first maturity**	< 2 years	2—10 years	> 10 years
	ES2	**Trophic level**	Less than 2	Between 2 and 4	Higher than 4
Resource Adaptive capacity	EA1	**Geographic range**	Latitudinal range < 45º	Latitudinal range between 45º and 90º	Latitudinal range > 90º
	EA2	**Resilience to fisheries**	Vulnerability score > = 66	Vulnerability score between 33 and 66	Vulnerability score < = 33

The final step in quantifying the social hazard dimension involves measuring resource user hazards by combining two indicators: storms and sea-level rise (indicators SH2 and SH3 in Table 1). To estimate the value of these indicators, we utilized questions from the survey (refer to Table 1 column Question/Data) to gather local ecological knowledge (Reyes‐García et al., 2016; Rosenzweig & Neofotis, 2013; Salgueiro-Otero & Ojea, 2020). These questions were directed to detect the occurrence of the climate drivers in the areas where the social community engages in fishing.^4^ We acknowledge, however, that the question used to measure the indicator of social hazard for “storminess” did not explicitly imply perceived increase in storms.

#### Quantifying Social Exposure

To quantify social exposure we used two indicators, namely exposure to sea level rise (coded SE1 in Table 1) and exposure to increasing storminess (coded SE2 in Table 1). Each indicator was determined based on responses obtained from our survey instrument. To calculate the proportion of households in the community that perceive exposure to sea level rise, we asked respondents if they had noticed any shoreline erosion in their area. Likewise, we calculated the proportion of households that perceive exposure to increasing storminess by asking respondents if they were directly impacted by a cyclone or large storm. Our approach to measuring these indicators relied on gathering local ecological knowledge, as we specifically drew on the perceptions of local communities. This is because the observations of the exposure to climate change by members of local communities serve as an essential data source for characterizing the local environment, especially when physical climate data is potentially lacking (García-del-Amo et al., 2020; Rosenzweig & Neofotis, 2013).

In summary for social hazard and social exposure, we assessed social hazard by measuring how resource users perceive the presence of sea-level rise and storms. On the other hand, we assessed social exposure by measuring how resource users perceive the impacts of their exposure to both sea-level rise and storms.

#### Quantifying social sensitivity

We utilized a set of four indicators to quantitatively assess social sensitivity to climate change. Namely, sensitivity to sea level rise, sensitivity to increasing storminess, direct dependence, and indirect dependence. The first two indicators were designed to capture the degree to which communities are sensitive to climate-related hazards, while the latter two were intended to measure the extent of their reliance on fish as a source of food and income. To assess the sensitivity to sea level rise, we utilized participant responses to the question: Have you observed any changes in your livelihood due to shoreline erosion? (See Table 2 column Question/Data). To evaluate the sensitivity to increasing storminess, we employed two questions from the survey instrument. Specifically, we inquired about the extent of damage caused by cyclones or large storms to participants’ households, as well as the number of fishing days lost to such events within the past year.

The direct dependence indicator was further subdivided into three sub-indicators: the dependence equation, nutrition indicator, and personal perception (SS3, SS4, and SS5, respectively). We adapted the dependence equation indicator from prior assessments (Barange et al., 2018), taking into account the available data from our survey. Specifically, the dependence equation was defined as follows:

4 $$Direct\;Dependence=\frac{DWF}{TDWP}$$

where *DWF* denotes days per week consuming fish and *TDWP* denotes total days per week consuming protein, including fish or any other animal protein. The sub-indicator of nutrition aims to quantify the protein content supplied by the functional seafood groups consumed by individual communities. To achieve this, we obtained values (in g/100 g) from peer-reviewed publications for a minimum of five fish species within each of the most frequently consumed functional seafood groups, as defined previously. We then applied weights to these values for each community based on the proportion of each functional seafood group consumed. The third sub-indicator, personal perception, was assessed using fishers’ responses to a question regarding their confidence in their ability to provide for their families without fishing (see Table 2 column Question/Data).

The final measure of social sensitivity is the indirect dependence indicator, which comprises three sub-indicators: income dependence, employment dependence, and wealth dependence (coded as SS6, SS7, and SS8 in Table 2, respectively). Income dependence was assessed by querying fishers about the proportion of their household income derived from fishing. Employment dependence was gauged by asking how critical fishing is as an economic activity in their community. Wealth dependence was evaluated by inquiring about the likelihood of fishers having to sell their homes in case fishing fails.

#### Social adaptive capacity

To assess the adaptive capacity of communities to changes in the abundance and distribution of resources provided by functional seafood groups we measure seven indicators, flexibility, assets, social organization, learning, equity, social cognition, and agency as found in the literature (Cinner & Barnes, 2019; Salgueiro-Otero & Ojea, 2020). The survey questions used to quantify each indicator, except for assets, and their respective sub-indicators are presented in Table 1 column Question/Data. The assets indicator was quantified using an Asset Wealth Index (Taylor et al., 2021) which was calculated based on responses to multiple survey questions related to various assets available to the communities, including their homes, livestock, savings, and other relevant factors.^5^ Although equity indicators are not commonly incorporated in vulnerability or risk assessments, the scientific literature recommends their inclusion for a comprehensive and informative evaluation of climate change adaptation. This is due to the fact that climate hazards can impact various individuals and groups within a community in distinct ways, influenced by variables such as socio-economic status, gender, and age. Incorporating equity indicators in the assessment process can provide valuable insights into the effectiveness of climate change adaptation measures, by ensuring that the needs of marginalized and vulnerable populations are accounted for. Due to limited available data, in order to develop an equity indicator, we used gender equity as a proxy for equity in general through responses to two specific gender-related questions: (1) To what extent do women hold leadership positions within the community? and (2) How equitable is women’s access to and control over their resources and livelihoods in comparison to men? These questions serve as valuable measures for assessing the level of gender equity within a given community.^6^

### Quantifying the CFSR index

We quantified the CFSR index by scaling, normalizing, and aggregating all indicators’ scores. To achieve this, a categorical scale comprising three values, low, intermediate, and high was considered, thus, converting all indicators to scores (see the SI for further insights on the scoring process). Following the conversion of indicators into scores, we have normalized every dimension, subdimension, sub-indicator, and indicator within the range of 0 to 1 using the equation presented below.

5 $${X}_{normalized}=\frac{{X}_{i}-{X}_{min}}{{X}_{max}-{X}_{min}}$$

where x_i_ denotes the value of the indicator. The observed minimum and maximum values are denoted by x_min_ and x_max_ respectively. Following normalization, equal weighting was assigned to all sub-indicators, and the scores were aggregated to obtain a risk dimension score. This approach is widely used in the literature on vulnerability and risk assessment to calculate scores for each dimension (Allison et al., 2009; Zebisch et al., 2021). After computing all risk dimensions, we calculated the CFSR index for each community using Eq. 1.

The quality of the CFSR index was evaluated by assessing the confidence level of the data used to calculate each indicator. A confidence level between 0 and 3 was assigned to each indicator, where a value of 3 represented the highest quality of data. Indicators that were calculated using data that was empirically measured, modeled, or directly observed were assigned a value of 3. For indicators that were estimated with limited data and a high degree of uncertainty, an intermediate value of 2 was assigned. Indicators that were assessed via expert elicitation or survey data were assigned a confidence value of 1. Finally, indicators that were not assessed due to a lack of data were assigned a value of 0; Table S4 in the SI summarizes data confidence level classification criteria. Once all indicators were assigned a confidence value, we calculated an overall confidence value for the final CFSR index, by community, by averaging across the indicators and dimensions, respectively.

## Results

### Social hazard dimension

The scores of resource hazard, exposure, sensitivity, and adaptive capacity of various functional seafood groups are presented in Table 3. The resources hazard score was the same for all functional seafood groups, 0.45 due to the proximity of the communities and the spatial scale of the data. The resource exposure varied across the species groups, with Small Pelagic Fish having the highest exposure, mostly driven by high sensitivity to changes in exposure to sea surface temperature and primary productivity. The resource sensitivity also differed across the species groups, with Large Pelagic Fish having the highest sensitivity (0.584), due to their higher trophic level, and Small Pelagic Fish having the lowest (0.250). The resource adaptive capacity of the species groups varied as well, with Small Pelagic Fish having the highest capacity (0.750) and Demersal species having the lowest (0.250). We used these values along with the proportion of seafood consumed by each community to calculate each community’s resource vulnerability.

**Table 3:** Ecological risk to resource scores. The normalized [0–1] score for each component of the ecological risk to the resource was calculated based on the average value of the families for each functional seafood group

	**Indicator**	**Functional Seafood Groups**				
Code		Coral Reef Fish	Small Pelagic Fish	Large Pelagic Fish	Cephalopods	Demersal spp.
EH1	SST	0.350	0.350	0.350	0.350	0.350
EH2	Primary Productivity	0.550	0.550	0.550	0.550	0.550
	average	0.450	0.450	0.450	0.450	0.450
EE1	Exposure to SST change	1.000	1.000	1.000	0.500	0.000
EE2	Exposure to PP change	0.500	1.000	0.333	0.750	0.000
	average	0.750	1.000	0.667	0.625	0.000
ES1	Age at maturity	0.375	0.000	0.167	0.000	0.500
ES2	Trophic Level	0.500	0.500	1.000	0.750	0.500
	average	0.438	0.250	0.584	0.375	0.500
EA1	Resilience to fisheries	0.625	1.000	0.333	0.250	0.000
EA2	Latitudinal range	0.500	0.500	0.833	1.000	0.500
	average	0.563	0.750	0.583	0.625	0.250

The results of the social hazard for each of the four communities, including its components ecological risk to resource and resources user hazard are presented in Table 4. Our results show moderate levels of ecological risk to resources across all communities. Interestingly, the resource user hazards show a wider variation, with values ranging from 0.532 to 0.849 with Unguja and Pemba having higher values than those of Mafia and Tanga, which are similar. Furthermore, the overall social hazard values indicate a moderate to high level of social vulnerability in these communities, with values ranging from 0.517 to 0.724, with Unguja presenting the highest social hazard index followed by Pemba, Tanga and Mafia, respectively.

### Social exposure, sensitivity, and adaptive capacity dimensions

Table 4 shows the results of the social exposure in the four fishing communities. The results show that Unguja has the highest exposure to sea level rise at 0.907, followed by Pemba and Mafia with 0.796 and 0.793, respectively. In contrast, Tanga has the lowest exposure to sea level rise at 0.574. Regarding storms, Pemba has the highest exposure with 0.324, while Mafia has the lowest exposure with 0.306. Furthermore, the overall social exposure index, which is a weighted average of exposure to sea level rise and storms, ranges from 0.480 in Tanga to 0.720 in Unguja, indicating considerable variability across the communities.

**Table 4:** Vulnerability scores by indicator and vulnerability components. The table presents the values for all CFSR indicators for the four study communities. It also includes the integrated score for all indicators (shown in bold), as well as the vulnerability score (AC-S). The final risk score is calculated using Eq. 1 and is subsequently normalized between 0 and 1. The scores representing the components of resource risk to the communities are based on the percentage of each functional seafood group consumed by the community. The table also includes a confidence level scale between 0 and 3, indicating the quality of data used

**Vulnerability component**	**Indicator**	**Code**	**Normalized (0–1)**				
			**Mafia**	**Pemba**	**Tanga**	**Unguja**	**Conf**
Resource Hazard	Combination (See Table 3)	EH1, EH2	0.450	0.450	0.450	0.450	3.000
Resource Exposure	Combination (See Table 3)	EE1, EE2	0.713	0.675	0.696	0.626	1.000
Resource Sensitivity	Combination (See Table 3)	ES1, ES2	0.415	0.448	0.441	0.466	2.800
Resource Adaptive Capacity	Combination (See Table 3)	EA1, EA2	0.635	0.604	0.640	0.649	3.000
**Resource Vulnerability**		**SH1**	**0.390**	**0.422**	**0.400**	**0.408**	2.900
**Ecological Risk to Resource**			**0.486**	**0.492**	**0.487**	**0.473**	1.950
Resources User Hazard	SLR Hazard	SH2	0.549	0.729	0.462	0.901	1.000
	Storm Hazard	SH3	0.516	0.771	0.615	0.798	1.000
**Resources Users Hazard index**			0.532	0.75	0.5385	0.849	1.00
**Social Hazard Index (Ecological Risk to Resource + Resource Users Hazard Index)**			**0.517**	**0.664**	**0.521**	**0.724**	1.500
Social Exposure	Exposure to SLR	SE1	0.793	0.796	0.574	0.907	1.000
	Exposure to Storms	SE2	0.306	0.324	0.344	0.338	1.000
**Social Exposure Index**			**0.550**	**0.590**	**0.480**	**0.720**	1.000
Social Sensitivity	Sensitivity to Storms	SS1	0.333	0.368	0.331	0.424	1.000
	Sensitivity to SLR	SS2	0.429	0.388	0.182	0.620	1.000
	Direct dependence	SS3	0.816	0.798	0.789	0.414	1.400
	Indirect dependence	SS4	0.580	0.580	0.550	0.489	1.000
**Social Sensitivity Index**			**0.580**	**0.588**	**0.519**	**0.648**	1.250
Social Adaptive Capacity	Flexibility	SA1	0.447	0.422	0.588	0.467	1.000
	Assets	SA2	0.362	0.234	0.390	0.374	1.258
	Social organization	SA3	0.634	0.463	0.608	0.611	1.000
	Learning	SA4	0.429	0.535	0.427	0.580	1.000
	Equity	SA5	0.625	0.520	0.600	0.546	1.000
	Social cognition	SA6	0.694	0.599	0.681	0.710	1.000
	Agency	SA7	0.783	0.803	0.771	0.833	1.000
**Social Adaptive Capacity Index**			**0.566**	**0.517**	**0.565**	**0.585**	1.154
**Vulnerability**			0.507	0.535	0.477	0.532	1.200
**Climate-Induced Food Security Risk (CFSR) Index**			**0.520**	**0.574**	**0.484**	**0.602**	1.293

Social sensitivity results indicate significant differences in the sensitivity of four coastal communities to storms, sea level rise, direct dependence, and indirect dependence. Specifically, the community of Unguja exhibited the highest sensitivity to both storm events (0.424) and sea level rise (0.620), while Tanga had the lowest sensitivity to sea level rise (0.182). Notably, the communities of Mafia and Pemba demonstrated similar sensitivities to storms (0.333 and 0.368, respectively) and sea level rise (0.429 and 0.388, respectively).

**Fig. 4:**
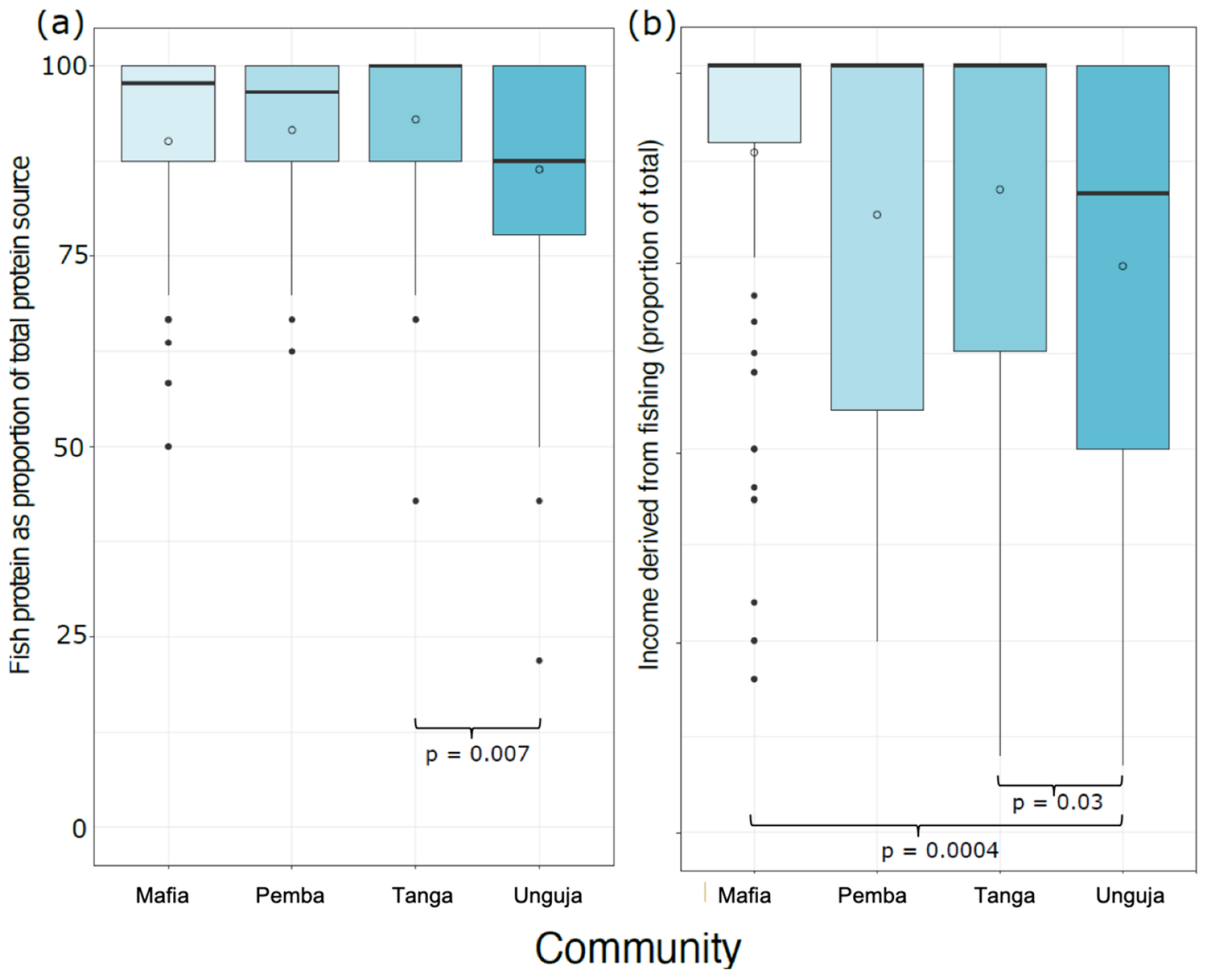
Community directly and indirectly dependence on fishing. Boxplots of two survey questions are presented for each community, which relate to their reliance on fishing. The first question, figure A, concerns the proportion of fish consumed as the primary source of protein in the community. The second question, figure B, pertains to the proportion of income earned from fishing. The figure displays significant differences between the communities, represented by the brackets and *p*-value

Table 4 also presents a comparison of the direct and indirect dependence on seafood. The data reveals that seafood is a significant source of protein for all communities, with each community consuming over 75% of their total protein from seafood. Notably, there were significant differences in protein consumption between Tanga and Unguja, with the latter consuming lower amounts (Fig. 4A). Furthermore, more than half of the total income of fishers in all communities comes from fishing (Fig. 4B). In Mafia, a striking 85% of fishers’ total income is derived from fishing, which is significantly higher than that of Unguja. Tanga and Unguja also differ significantly in their income derived from fishing, with Tanga showing higher levels (Fig. 4B). We used results from Fig. 4 to calculate the direct and indirect dependence indicators. The results reveal that the community of Mafia exhibits the highest direct dependence on their local ecosystem, with a score of 0.816, followed closely by Pemba and Tanga with scores of 0.798 and 0.789, respectively. However, when considering both direct and indirect dependence, the overall Social Sensitivity Index of these communities differs, with Pemba exhibiting the highest score of 0.588, followed by Unguja with 0.648, while Tanga and Mafia scored 0.519 and 0.58, respectively.

**Fig. 5:**
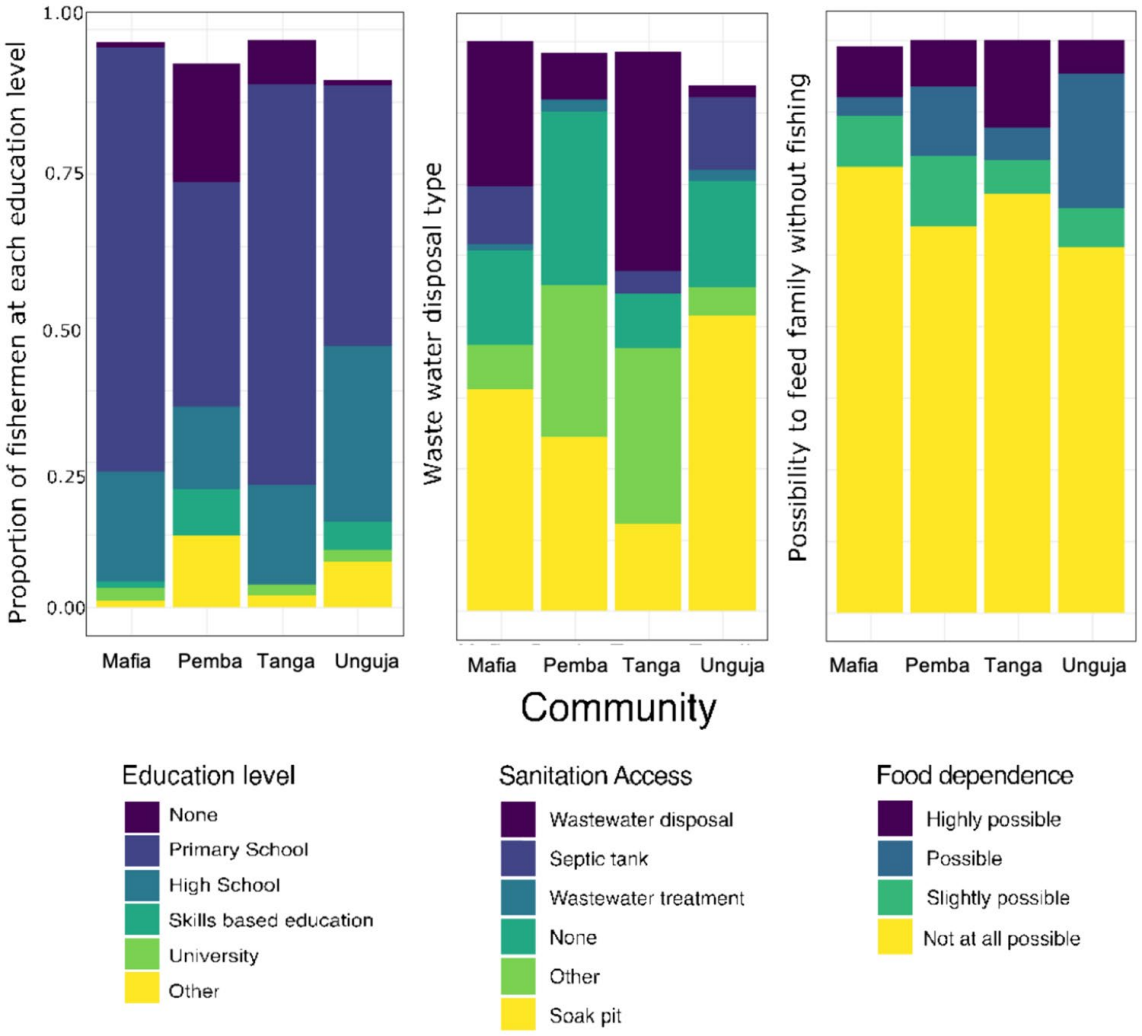
Education, wastewater disposal, and food security by communities. Each plot displays questionnaire responses concerning three questions regarding the social aspects of the community. Question A represents the education level of the fishers in the community (left plot). Question B pertains to the available wastewater disposal types in the communities (center plot). Question C explores the possibility of families within the community being able to feed themselves without relying on fishing (right plot)

Figure 5 illustrates the responses to three questions related to social adaptive capacity indicators. Figure 5A depicts the educational attainment of fishers across all communities, revealing that the majority possess primary school education, while less than 3% have completed university-level studies. Notably, nearly one-quarter of fishers in Pemba lack any formal education, indicating generally low educational levels among these communities. In Fig. 5B, the level of sanitation access is presented as a proxy measure of community assets and infrastructure (Brooks et al., 2005). The data show that in Pemba, Mafia, and Unguja, over 75% of the population has access to only rudimentary forms of sanitation disposal, such as a soak pit, no sanitation disposal, or "other." Some residents of Unguja and Mafia have access to a septic water tank, while Tanga displays the highest levels of access to modern sanitation disposal, with between 25–50% of the community possessing wastewater disposal facilities. The pivotal role of fishing in the livelihoods of these communities is conveyed in Fig. 5C, which indicates that more than 63% of fishers across all community’s report that fishing is essential for feeding their families.

Table 4 shows that while certain adaptive capacity indicators show substantial variation across communities, the overall adaptive capacity of these communities is relatively similar. Among the different indicators, social cognition is consistently high across all communities, with Unguja exhibiting the highest score of 0.710, followed closely by Mafia at 0.694. Meanwhile, assets appear to be the lowest-scoring indicator, with all communities scoring below 0.4. Flexibility also shows substantial variation, with Tanga having the highest score of 0.588, while Pemba has the lowest score of 0.422. In contrast, agency, which measures the ability to act and make decisions, exhibits the least variability, with all communities scoring above 0.77. Overall, the social adaptive capacity index, the weighted sum of the seven indicators, shows that all communities have relatively similar scores, ranging from 0.517 for Pemba to 0.566 for Mafia.

### The CFSR index

**Fig. 6:**
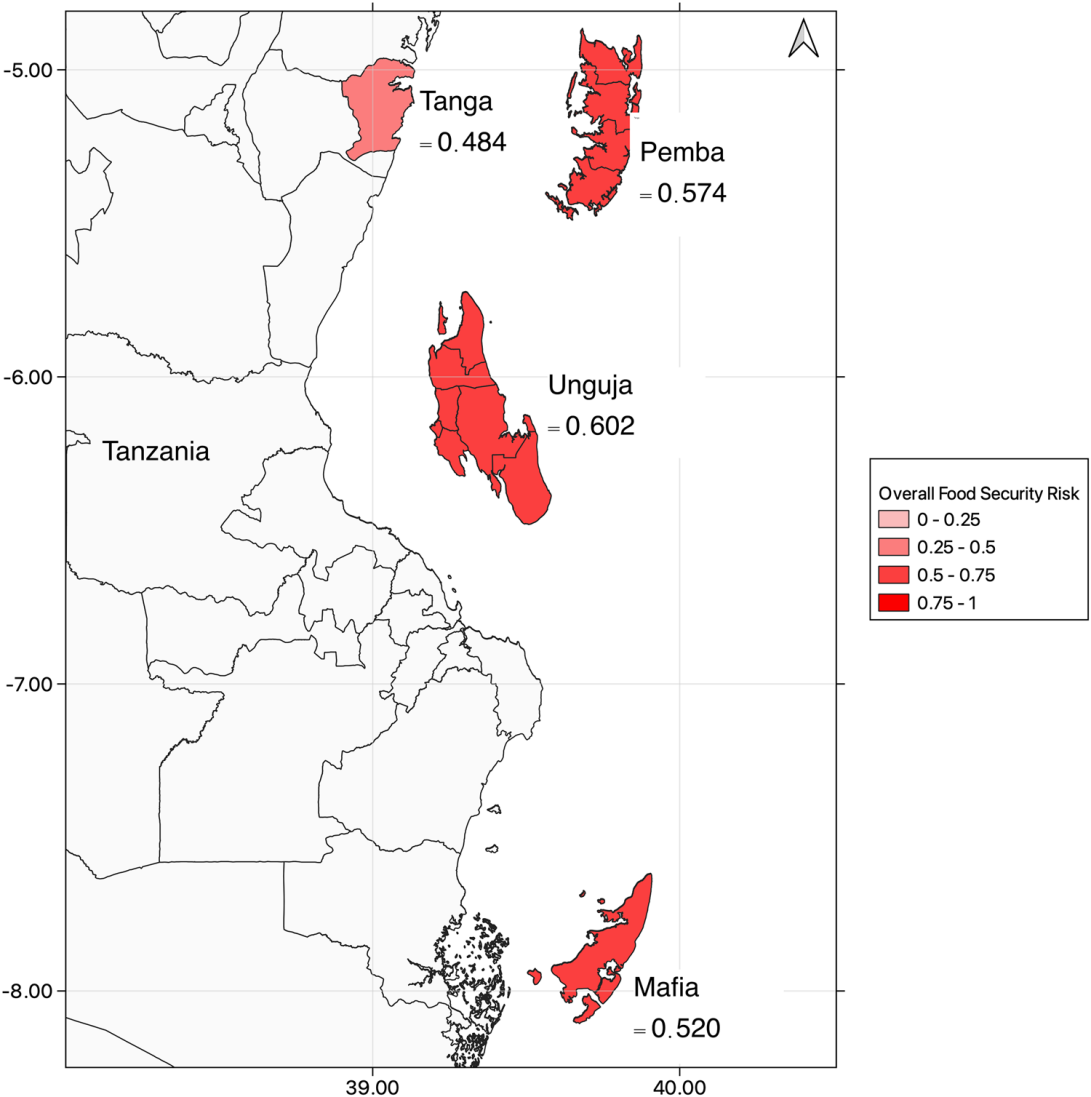
Overall scores of climate-induced food security risk to the four Tanzanian communities

The above results indicate that the highest social hazard index was found in Unguja, with a value of 0.724, followed by Pemba with 0.664. In terms of social exposure index, Unguja also showed the highest value of 0.720, while Pemba demonstrated the lowest value of 0.590. Furthermore, the social sensitivity index was the highest in Unguja with a score of 0.424, while the lowest score was found in Mafia, with a value of 0.333. In contrast, the social adaptive capacity dimension showed the highest score in Tanga, with a value of 0.565. Using our CFSR framework and Eq. 1 our results show that communities’ risks are moderate to high with different aspects driving the final score. Most at-risk communities identified are Pemba and Unguja (0.574 and 0.602, respectively). Mafia’s community risk score was 0.520, and lastly, Tanga had a food security risk score of 0.484 (Fig. 6). Tanga scores were lower in comparison, driven by a lower overall exposure and sensitivity than the other three communities to climate impacts on their food security. Table 4 reveals the confidence levels of all data sources used to compute the indicators, dimensions, and the CFSR index. The confidence levels demonstrate that the data sources with high confidence levels are related to the indicators utilized to calculate the components of ecological risk to resources, whereas the rest of the sources have an average value of 1. These results are primarily due to the utilization of survey instrument data to calculate the exposure, sensitivity, and adaptive capacity indicators.

## Discussion

Food security is a crucial issue in the face of climate change, and a local understanding of communities’ susceptibility to its impact is necessary for effective management and policy interventions. In this study, we developed a risk assessment framework that focuses specifically on the food security implications of climate change on SSF communities. By operationalizing this framework to a case study of four regions in Tanzania, we found that although all communities had similar overall food security risk scores (mid to high risk levels), the key drivers of risk differed between them. The results enable us to rank communities based on their risk levels and prioritize interventions by targeting specific contributors to risk.

**Fig. 7:**
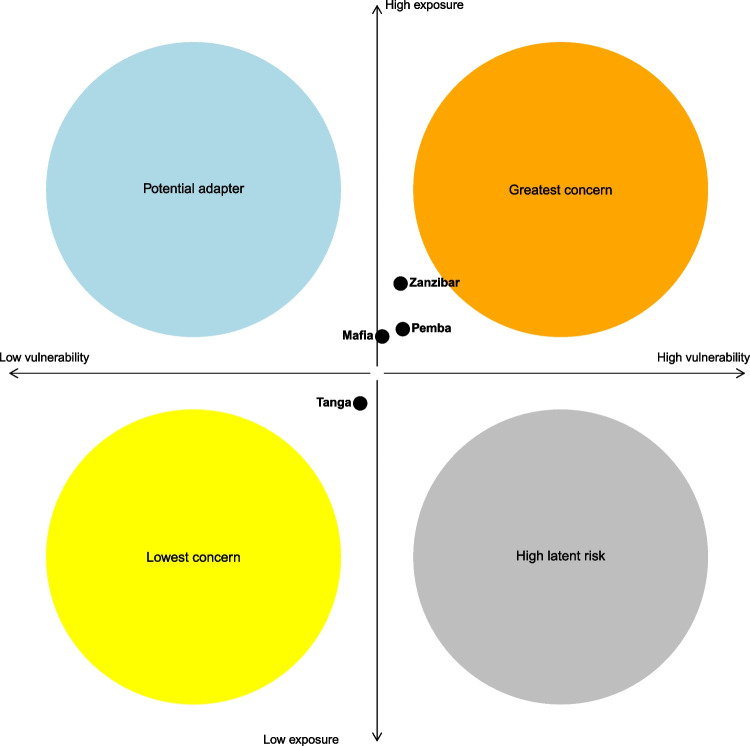
Communities risk profiles. Risk profiles depicting the inherent risk components based on scores of vulnerability and exposure. Based on Thiault et al. (2019)

We analyzed the different components of risk separately to determine causal drivers and identify effective intervention strategies. To do this, we followed an approach that combines components of vulnerability to create profiles that highlight the most applicable interventions to reduce the risk. The interventions are discussed below. Thiault et al. (2019) suggest that communities with high hazard, exposure, and vulnerability scores require interventions to reduce resource dependency and build adaptive capacity. On the other hand, communities with high vulnerability but less exposure to hazards would benefit from measures to increase their adaptive capacity. Finally, communities that are highly exposed to hazards but have low vulnerability are potential adaptors that would benefit from reducing their dependency on resources. Our study estimated the components of risk and framed them within the context of these profiles to classify the communities’ risk and envisage potential interventions (Fig. 7).

The findings of this study indicate that high social exposure across all communities may indicate the presence of functional seafood groups in either the "greatest concern" or "potential adapters" profiles, both of which require policy interventions to reduce risk. The vulnerability, calculated as adaptive capacity minus sensitivity, is moderate across all communities (ranging from 0.390 to 0.422). To mitigate the risk, methods such as enhancing the intrinsic resilience of the resources or reducing exposure can be employed (Thiault et al., 2019). However, reducing exposure to climate hazards is not always feasible, and thus enhancing resilience is likely to be the most effective approach. Increasing resilience can be achieved by reducing fishing pressure as an additional stressor on the resources (Sumaila & Tai, 2020). One potential eco-centric policy intervention would be to establish a multi-use marine area with a no-take zone as part of marine spatial planning in Pemba. This approach would allow coral reefs and associated reef fish to regenerate, ultimately increasing fish stock availability locally and in adjacent areas, thereby reducing food insecurity resulting from climate-driven changes (Sala et al., 2021). However, such a policy intervention may be less crucial in Unguja where a different policy intervention, such as Individual Transfer Quotas (ITQs)—which have been shown to be effective in maintaining sustainability of highly migratory species (Edvardsson et al., 2018)—would benefit communities reliant on pelagic stocks. In any case, for any intervention, it is essential to ensure local fishers’ rights over their resources in order to increase stewardship and decrease future vulnerabilities (Ojea et al., 2017) In parallel, engaging communities in participatory planning processes of co-creation is critical to guarantee just and equitable adaptations and transformations towards food security risk reduction (Cooley et al., 2022).

Improving the sustainability of fishing techniques and fishing in all communities would also be fundamental for increasing the resilience of the fish species. Along the Tanzanian coast, particularly the mainland coastline of Tanga, explosive fishing has been reported (Samoilys & Kanyange, 2008), which not only kills the targeted species but also destroys habitats of many other species and strongly impairs recruitment. Thus, controlling these detrimental techniques would be a fundamental first step toward increasing the resilience of key food resources and ensuring food security in the mid-term.

The three island communities of Mafia, Pemba, and Unguja present risk profiles of "greatest concern" as defined by Thiault et al. (2019); see Fig. 7. This is due to their moderate to high scores of hazard and exposure and moderate to high scores of vulnerability. Unguja, in particular, presents high scores of hazard and exposure, high sensitivity, but reasonably high adaptive capacity. In contrast, Tanga displays lower levels of overall risk across the components. Tanga is the only mainland community in the study, and it appears as the lowest concern community in relation with the others, which may be a key reason for the results found. Mainland versus island fishers are likely to face slightly different challenges, for instance, island fishers (Benansio & Jiddawi, 2016) have expressed that fishing is their primary occupation due to the lack of land suitable for farming and the lack of other income-generating activities. However, as seen in Fig. 7, the risk values from Tanga still place the community on the edges of the profile and risk result is not distant from the other communities. Therefore, the result should not be interpreted as if interventions and policy evaluations for improving food security under climate risk are not needed in Tanga.

The work of Thiault et al. (2019) envisages two types of actions that can be taken to reduce food security risk over communities in the category of great concern and potential adapters: reducing resource dependency and building adaptive capacity. Resource dependency was found to be very high across the four communities. Livelihood-focused measures could potentially be appropriate to diversify occupations, such as tourism, and primary food sources through aquaculture or freshwater fishing, for example. In developing regions, such as the Western Indian Ocean (WIO), aquaculture has not been developed sufficiently due to limited technology and investments (Golden et al., 2016; Hall et al., 2013). It is also unrealistic that fishers could easily switch to aquaculture as significant resources would need to be established, in addition to a technology transfer and consumer acceptance. Climate impacts on aquaculture would also need to be thoroughly examined and could imply an additional risk. However, this could represent an investment opportunity for governments to secure an alternative food production avenue. These interventions could cause conflicts with fisheries creating further challenges for the communities. Spending less time fishing could result in a loss of access to fish as a food supply, which could have a negative impact on their food security. Therefore, reducing the dependence of communities on fishing is a complex issue that requires further attention.

Effective strategies for promoting both social and ecological benefits may be achieved by building adaptive capacity (Wright et al., 2016). For instance, interventions in Pemba should aim to increase assets and social organization by investing in community infrastructure and management, considering the low social adaptive capacity in the area. Similarly, low scores for learning in Tanga and Mafia suggest the need for further investment in education. Across all communities, the urgency for increased investment in community infrastructure is indicated by the very low scores of assets. One significant asset for local fishers is the type of boats used, which are mainly dug-out canoes and non-motorized boats, as revealed by survey answers and supported by local studies (Makame & Salum, 2021). Investment in social aspects such as education, infrastructure improvement, and the adoption of specific management rules is essential for Tanzanian communities to enhance adaptive capacity in the fishing communities and facilitate adaptation to the impacts of climate change.

While our study suggests that the CFSR is a powerful tool for assessing climate-induced food security risks while taking local conditions into account, there are some limitations to our approach. One clear limitation is the lack of granularity of our observed data. We defined the climate data quality with the highest score of 3, despite the fact that we used average values across communities—due to proximity of islands and mobility of fishers and seafood. This approach might not fully capture the nuanced variations within individual communities and we would suggest for future studies to utilise more fine-scale data. Another limitation which is acknowledged is that all dimensions of risk are given equal weighting, which assumes that all vulnerability indicators are equally important. Alternatively, one can use expert elicitation to provide subjective judgments on the importance of each vulnerability indicator or apply Principal Component Analysis to identify the most important factors contributing to vulnerability based on data covariance. However, principal components may not be easy to interpret for our food security index purposes, and we leave these considerations for future developments. Our study takes a snapshot of the current state of the four communities of interest. We recognize that risk is not static; it changes over time as communities adapt and as environmental and economic conditions evolve. Finally, we recognize that translating our findings and the suggested solutions into effective policy and action is a challenge. This will depend on the willingness of stakeholders and the mechanisms for integrating these findings into the practical decision-making process.

## Conclusions

Our approach suggests the potential for using existing information and local knowledge in developing countries like Tanzania to assess climate change impacts on food security at a community level. While the study utilized average values for climate data, future research could benefit from more fine-scale data to further refine the risk assessments.

The implications of the study are three-fold; firstly, the CSRF we developed provides a mechanism to obtain a detailed understanding of food security risks due to climate change at a local level, by integrating social, economic and ecological aspects. Secondly, the integration of multiple data sources (ranging from local ecological knowledge to observed climate data) is necessary to measure the various dimensions of climate-induced food security risk. Finally, we demonstrated that it is vital to apply a refined lens in understanding community challenges and risks as an evidentiary basis for effective management and policy interventions.

Overall, this study underscores the importance of localized, tailored interventions in managing the food security risks posed by climate change, particularly in small-scale fishing communities Nuanced recognition of risk components is needed to inform both policy interventions and adaptive management responses. The framework developed can provide a tool which can be shaped to deliver the differentiated understanding that is required for policies or interventions that relate to context specific challenges rather than broader brushstroke interventions that may miss crucial differences between communities. Other regions could adapt and apply the framework in their own contexts. Significantly, the results demonstrated that highly contrasting policy responses (e.g. individual transfer quotas versus marine protected areas) could be relevant even within a small geographic area, due to the unique risk characteristics of each community. In conclusion, our findings reveal the necessity for community-specific data as a basis to inform interventions in the face of climate change.

## Supplementary Information

Below is the link to the electronic supplementary material.

Supplementary file1 (DOCX 50 KB)

## Data Availability

The authors confirm that the data supporting the findings of this study are available within the article, its supplementary materials, and cited literature.

## Footnotes

1 https://www.globcolour.info/.

2 www.fishbase.org.

3 The resource consumption of Mafia is mainly comprised of coral reef fish, accounting for 71% of their total consumption. In Pemba and Tanga, more than half of the fish consumed is coral reef fish, while a considerable proportion of their consumption consists of large pelagic fish. Conversely, in Unguja, large pelagic fish is the predominant type of fish consumed, accounting for 50% of their total consumption. Small pelagic fish are consumed across all communities, while the consumption of demersal species and cephalopods is relatively low, comprising only 4% or less of the total consumption in all communities except for Pemba, where it accounts for 8% of their total consumption.

4 The survey responses reveal that more than 60% of fishers across all regions reported experiencing severe storms and changes to their shoreline in the last five years.

5 See Taylor et al. (2021) for a detailed description of the assets indicator calculation.

6 We however need to acknowledge that survey respondents are all men and therefore the voices of the women have not been collected in the available data and a gender bias may be present.
