# Supplementary material for: A new framework on climate-induced food-security risk for small-scale fishing communities in Tanzania
Source: Food Secur. 2024 Jul 19;16(5):1125–45. doi: 10.1007/s12571-024-01472-x (PMC11489249; doi:10.1007/s12571-024-01472-x)
Supplement: Supplementary file 1 — Supplementary file1 (DOCX 50 KB) [file 12571_2024_1472_MOESM1_ESM.docx]

Supplementary Information for

Article title: A new framework on Climate-Induced Food Security Risk for Small-Scale Fishing Communities in Tanzania.

Journal: Food Security

Authors: Lara Paige Brodie^1^, Smit Vasquez Caballero^1,2^, Elena Ojea^1^, Sarah FW Taylor^3^, Michael Roberts^3,4^, Patrick Vianello^4^, Narriman Jiddawi^5^, Shankar Aswani^6^, Juan Bueno-Pardo^1*^

^1^ Centro de Investigación Mariña (CIM), Universidade de Vigo, Future Oceans Lab, Campus Lagoas Marcosende, 36310 Vigo, Spain.

^2^ Center for Applied Economics and Strategy, RTI International, Research Triangle Park, NC 27709, USA

^3^ National Oceanography Centre, Southampton, SO14 3ZH, United Kingdom

^4^ Nelson Mandela University, Port Elizabeth, South Africa

^5^ Institute of Marine Sciences, UDSM, Tanzania

^6^ Rhodes University, Grahamstown, South Africa

* Corresponding author: juan.bueno@uvigo.gal

**Proportion of fish consumed by each community**

To determine the ecological risk indicator, we examined the main food sources, particularly the primary types of fish consumed by households in different communities. These types were sorted into five functional groups, which included coral reef fish, small pelagic fish, large pelagic fish, demersal species, and cephalopods. The groupings were created by combining various fish families, as per FishBase (www.Fishbase.org). Subsequently, we computed the proportion of each functional group consumed by each community, based on the frequency with which a particular group was reported as the most commonly consumed. Table S1 presents the proportion of each fish family consumed by a particular community. For instance, in the Pemba community, Lethrinidae (a type of coral reef fish) accounts for 68% of the total coral reef fish consumed, while Engraulidae (a type of small pelagic fish) represents 78% of the total small pelagic fish consumed. Table S2 shows the proportion of different functional groups of fish consumed by each community. The table shows that in Mafia, the most commonly consumed functional group is coral reef fish, representing 71% of the fish consumed, followed by large pelagic fish at 21%. Small pelagic fish and cephalopods make up a smaller proportion of the consumed fish. In Pemba, coral reef fish are also the most commonly consumed fish group, but they represent a lower proportion of 47%, while large pelagic fish make up a higher proportion of 25%. Small pelagic fish and demersal species are also consumed in Pemba. In Tanga, the proportions of coral reef fish and small pelagic fish are similar at 50% and 13%, respectively, while large pelagic fish are consumed the most, representing 35% of the fish consumed. In Unguja, the most commonly consumed fish group is large pelagic fish, representing 50% of the fish consumed, followed by coral reef fish at 38%. Demersal species and cephalopods are consumed in much smaller proportions in Unguja.

| Table S1 Proportion of each family consumed within each functional group by community | | | | | |
| --- | --- | --- | --- | --- | --- |
|  |  | Community | | | |
| Functional Group | Family | Mafia | Pemba | Tanga | Unguja |
| Coral Reef Fish | Lethrinidae | 0.33 | 0.68 | 0.44 | 0.33 |
|  | Carangidae | 0.27 | 0.05 | 0.33 | 0.28 |
|  | Siganidae | 0.40 | 0.00 | 0.17 | 0.13 |
|  | Lutjanidae | 0.00 | 0.26 | 0.06 | 0.26 |
| Small Pelagic Fish | Engraulidae | 1.00 | 0.78 | 1.00 | 0.70 |
|  | Clupidae | 0.00 | 0.22 | 0.00 | 0.30 |
| Large Pelagic Fish | Scombridae | 0.89 | 1.00 | 1.00 | 1.00 |
|  | Rachycentridae | 0.05 | 0.00 | 0.00 | 0.00 |
|  | Xiphiidae | 0.05 | 0.00 | 0.00 | 0.00 |
| Demersal Species | Eels, rays, skates | 0.00 | 1.00 | 0.00 | 1.00 |
| Cephalopods | Octopodidae | 0.50 | 1.00 | 1.00 | 0.00 |
|  | Squid | 0.50 | 0.00 | 0.00 | 1.00 |

| Table S2. Proportion of the functional groups consumed at each community | | | | |
| --- | --- | --- | --- | --- |
|  | Community | | | |
|  | Mafia | Pemba | Tanga | Unguja |
| Coral Reef Fish | 0.71 | 0.47 | 0.50 | 0.38 |
| Small Pelagic Fish | 0.06 | 0.17 | 0.13 | 0.09 |
| Large Pelagic Fish | 0.21 | 0.25 | 0.35 | 0.50 |
| Demersal Species | 0.00 | 0.08 | 0.00 | 0.02 |
| Cephalopods | 0.02 | 0.04 | 0.02 | 0.02 |

**Functional groups and fish families ecological risk quantification**

We obtained indicators characterizing the life history of the majority of species within each fish family using information from Fishbase.org. To quantify the exposure, sensitivity, and adaptive capacity dimensions per functional group, we employed a categorical scale with three values: low, intermediate, and high. Table S3 displays the categorical values of the indicators estimated for each functional group and dimension.

| Table S3 Fish families categorical values by dimension and indicators | | | | | | | |
| --- | --- | --- | --- | --- | --- | --- | --- |
|  | Dimension | Exposure |  | Sensitivity |  | Adaptive capacity | |
|  | Indicator | E1 | E2 | S1 | S2 | A1 | A2 |
|  | Indicator evaluation | Species occurrence/distribution | Species occurrence/distribution | Species' life history traits | Species' life history traits | Vulnerability to fisheries (Cheung et al. 2005) | Latitudinal range occupied |
| Coral Reef Fish | Lethrinidae | High | Medium | Medium | Medium | Medium | Medium |
|  | Carangidae | High | Medium | Medium | Medium | Medium | Medium |
|  | Siganidae | High | Medium | Low | Medium | High | Medium |
|  | Lutjanidae | High | Medium | Medium | Medium | Medium | Medium |
| Small Pelagic Fish | Engraulidae | High | High | Low | Medium | High | Medium |
|  | Clupeidae | High | High | Low | Medium | High | Medium |
| Large Pelagic Fish | Scombridae | High | Low | Low | High | Medium | High |
|  | Rachycentridae | High | Low | Low | High | Medium | Medium |
|  | Xiphiidae | High | High | Medium | High | Low | High |
| Cephalopods | Octopodidae | Medium | High | Low | Medium | Low | High |
|  | Squid spp. | Medium | Medium | Low | High | Medium | High |
| Demersals | Demersal spp. | Low | Low | Medium | Medium | Low | Medium |
| Notes.  Indicator notation: E1. Exposure to SST change. E2. Exposure to Ocean primary production change. S1. Age at maturity S2. Trophic level A1: Resilience to fisheries. A2. Extension of occurrence range  Indicator evaluation and their categorization:  Species occurrence/distribution: High: Coral-reef-associated species or species with pelagic adults, larvae, and eggs. Medium: Demersal species occurring mainly above 50 m depth. Low: Demersal species occurring mainly below 50 m depth  Species occurrence/distribution: High: Filter-feeding species.Medium: Feeding on macroscopic invertebrates, fish larvae, algae grazers, etc. Low: Higher level predators.  Species' life history traits: High: >10 years. Medium: 2 - 10 years. Low: < 2 years  Species' life history traits: High: >4. Medium: 2 - 4. Low: < 2.  Vulnerability to fisheries (Cheung et al. 2005): High: Vulnerability score <= 33. Medium: Vulnerability score between 33 and 66. Low: Vulnerability score >= 66.  Latitudinal range occupied High: >90º latitudinal range. Medium: 45-90º latitudinal range. Low: <45º latitudinal range. | | | | | | | |

**Scoring Values**

All indicators were evaluated using a categorical scale with three values: low, intermediate, and high. Some indicators were obtained directly in this scale (e.g., expert elicitation indicators) from the data source used to evaluate them, while others required some posterior adjustment. For the indicators estimated using the questionnaire, standardization was done by converting the Likert values into: 1 = "High," 2/3 = "Moderate," and 4 = "Low." This process was adapted for each question and scale to ensure that the values corresponded to the correct level of the risk component. For "Yes/No" answers, "Yes" was coded as "High sensitivity," and "No" as "Low sensitivity," depending on the nature and information provided by the question. For numerical answers, a threshold value based on national statistics (WHO, 2020) or the minimum and maximum values of the indicator itself was used. This method was applied to a few indicators, such as "life expectancy at birth," "the dependence equation," "nutrition indicator," and "number of markets to buy/sell fish"; see Table S4.

| Table S4: Threshold values used for normalizing sub-indicators | | | |
| --- | --- | --- | --- |
| **Normalization thresholds** | **Minimum value** | **Maximum value** | **Source** |
| Life expectancy at birth: | 50.7 | 84.3 | WHO, 2020 |
| Nutrition indicator: | 0 | 20.36 | Value of the most nutritious functional group |
| Number of markets to buy and sell fish | 0 | 3 | Based on number of markets indicated by fishers |
| All other questions | 1 | 3 | Heck et al., 2019 |

**Data confidence level criteria**

| Table S5: Confidence Interval Score Criteria | | | |
| --- | --- | --- | --- |
| **Score** | **Title** | **Description** | **Relevant data** |
| 3 | Adequate Data | The score is based on data which have been observed, modeled or empirically measured for the species in question and comes from a reputable source. | - Life history trait data taken from fishbase where there is information for all species  - Variation index: data based on models |
|  |  |  |  |
| 2 | Limited Data | The score is based on data which has a higher degree of uncertainty. The data used to score the attribute may be based on related or similar species, come from outside the study area or the reliability of the source may be limited. | - life history data for which data for less than half of the species are present |
| 1 | Expert Judgment | The attribute score reflects the expert judgment of the reviewer and is based on their general knowledge of the species, or related species, and their relative role in the ecosystem. | - Expert opinion data (i.e. fish exposure)  - Survey answers |
| 0 | No Data | No information to base an attribute score on. Very little is known about the species or related species and there is no basis for forming an expert opinion. |  |

| Table S6: Life history trait data obtained from Fishbase ([www.Fishbase.org](http://www.fishbase.org)) for all fish functional groups utilised, represented by each individual species within each functional group. | | | | | | | |
| --- | --- | --- | --- | --- | --- | --- | --- |
| **Functional Group** | **Scientific Name** | **Family** | **Trophic level** | **Age at first maturity** | **Latitudinal Range** | **IUCN Red list category** | **Vulnerability score** |
| Small Pelagic Fish | Amblygaster sirm | Clupeidae | 2.9 | 0.6 | 63 | LC | 17 |
|  | Herklotsichthys punctatus | Clupeidae | 3.6 | 0.6 | 49 | LC | 10 |
|  | Herklotsichthys quadrimaculatus | Clupeidae | 2.8 | 0.4 | 72 | LC | 15 |
|  | Herklotsichthys spilurus | Clupeidae | 2.6 | 0.6 | 34 | LC | 10 |
|  | Hilsa kelee | Clupeidae | 2.8 | 0.7 | 43 | LC | 18 |
|  | Spratelloides gracilis | Clupeidae | 3 | 0.2 | 63 | LC | 10 |
|  | Encrasicholina devisi | Engraulidae | 3.6 | 0.4 | 61 | LC | 10 |
|  | Encrasicholina heteroloba | Engraulidae | 2.9 | 0.4 | 58 | LC | 10 |
|  | Encrasicholina intermedia | Engraulidae | 3.3 | 0.7 | 52 | DD | 11 |
|  | Encrasicholina punctifer | Engraulidae | 3.3 | 0.7 | 77 | LC | 12 |
|  | Stolephorus commersonnii | Engraulidae | 3.1 | 0.9 | 51 | LC | 14 |
|  | Stolephorus indicus | Engraulidae | 3.6 | 0.8 | 67 | LC | 13 |
|  | Thryssa baelama | Engraulidae | 2.9 | 1.2 | 56 | LC | 23 |
|  | Thryssa setirostris | Engraulidae | 3.3 | 1.9 | 68 | LC | 29 |
|  | Thryssa vitrirostris | Engraulidae | 3.4 | 1.3 | 71 | LC | 24 |
| Coral Reef Fish | Siganus argenteus | Siganidae | 2 | 1 | 60 | LC | 22 |
|  | Siganus luridus | Siganidae | 2 | 3.5 | 70 | LC | 39 |
|  | Siganus stellatus | Siganidae | 2.7 | 1.1 | 35 | LC | 24 |
|  | Siganus sutor | Siganidae | 2.2 | 1.1 | 36 | LC | 26 |
|  | Gnathodentex aureolineatus | Lethrinidae | 3.5 | 1.8 | 60 | LC | 30 |
|  | Gymnocranius elongatus | Lethrinidae | 4 | 2.1 | 60 | LC | 33 |
|  | Gymnocranius grandoculis | Lethrinidae | 3.4 | 2.9 | 63 | LC | 42 |
|  | Lethrinus borbonicus | Lethrinidae | 3.7 | 0.9 | 60 | LC | 21 |
|  | Lethrinus conchyliatus | Lethrinidae | 3.6 | 3.7 | 36 | LC | 53 |
|  | Lethrinus crocineus | Lethrinidae | 3.9 | 3.1 | 38 | LC | 44 |
|  | Lethrinus erythracanthus | Lethrinidae | 3.4 | 3.3 | 53 | LC | 46 |
|  | Lethrinus erythropterus | Lethrinidae | 3.8 | 2.8 | 50 | LC | 38 |
|  | Lethrinus harak | Lethrinidae | 3.8 | 1.4 | 64 | LC | 27 |
|  | Lethrinus lentjan | Lethrinidae | 3.9 | 1 | 67 | LC | 25 |
|  | Lethrinus mahsena | Lethrinidae | 4 | 6.5 | 54 | EN | 65 |
|  | Lethrinus microdon | Lethrinidae | 3.8 | 3.2 | 55 | LC | 46 |
|  | Lethrinus nebulosus | Lethrinidae | 3.8 | 3.1 | 68 | LC | 46 |
|  | Lethrinus obsoletus | Lethrinidae | 3.8 | 1.9 | 53 | LC | 33 |
|  | Lethrinus olivaceus | Lethrinidae | 3.4 | 2.5 | 68 | LC | 40 |
|  | Lethrinus rubrioperculatus | Lethrinidae | 3.6 | 1.7 | 69 | LC | 31 |
|  | Lethrinus variegatus | Lethrinidae | 4.2 | 1.6 | 65 | LC | 26 |
|  | Lethrinus xanthochilus | Lethrinidae | 4.3 | 4.6 | 59 | LC | 57 |
|  | Monotaxis grandoculis | Lethrinidae | 4.4 | 3.1 | 65 | LC | 44 |
|  | Wattsia mossambica | Lethrinidae | 4 | 1.3 | 69 | LC | 28 |
|  | Atule mate | Carangidae | 4.3 | 0.9 | 70 | LC | 19 |
|  | Carangoides armatus | Carangidae | 4.4 | 2.1 | 62 | LC | 35 |
|  | Carangoides chrysophrys | Carangidae | 4.1 | 2.6 | 72 | LC | 41 |
|  | Carangoides coeruleopinnatus | Carangidae | 3.9 | 1.6 | 67 | LC | 29 |
|  | Carangoides dinema | Carangidae | 4.5 | 1.1 | 54 | LC | 32 |
|  | Carangoides ferdau | Carangidae | 3.7 | 3 | 72 | LC | 44 |
|  | Carangoides fulvoguttatus | Carangidae | 4.2 | 4 | 66 | LC | 66 |
|  | Carangoides gymnostethus | Carangidae | 4.5 | 3 | 64 | LC | 47 |
|  | Carangoides malabaricus | Carangidae | 4 | 0.9 | 80 | LC | 25 |
|  | Carangoides oblongus | Carangidae | 4.5 | 1.7 |  | LC | 31 |
|  | Carangoides orthogrammus | Carangidae | 4.1 | 2.5 | 69 | LC | 40 |
|  | Caranx heberi | Carangidae | 3.4 | 2.8 | 73 | LC | 45 |
|  | Caranx ignobilis | Carangidae | 4 | 7 | 55 | LC | 82 |
|  | Caranx melampygus | Carangidae | 3.4 | 3.3 | 70 | LC | 56 |
|  | Caranx papuensis | Carangidae | 3.7 | 1.1 | 65 | LC | 33 |
|  | Caranx sexfasciatus | Carangidae | 4.3 | 2.6 | 68 | LC | 45 |
|  | Caranx tille | Carangidae | 3.8 | 2.3 | 66 | LC | 38 |
|  | Decapterus kurroides | Carangidae | 3.9 | 1.1 | 64 | LC | 25 |
|  | Decapterus macarellus | Carangidae | 3.4 | 0.4 | 100 | LC | 20 |
|  | Decapterus macrosoma | Carangidae | 2.9 | 0.6 | 73 | LC | 17 |
|  | Decapterus russelli | Carangidae | 4.4 | 1.1 | 76 | LC | 24 |
|  | Elagatis bipinnulata | Carangidae | 4 | 2.5 | 85 | LC | 51 |
|  | Gnathanodon speciosus | Carangidae | 4.1 | 1.3 | 66 | LC | 38 |
|  | Megalaspis cordyla | Carangidae | 3.8 | 1 | 69 | LC | 29 |
|  | Naucrates ductor | Carangidae | 4.4 | 0.3 | 104 | LC | 24 |
|  | Parastromateus niger | Carangidae | 4.2 | 1.2 | 62 | LC | 30 |
|  | Scomberoides commersonnianus | Carangidae | 3.3 | 4.2 | 77 | LC | 64 |
|  | Scomberoides lysan | Carangidae | 3.6 | 1.9 | 55 | LC | 40 |
|  | Scomberoides tol | Carangidae | 3.2 | 0.7 | 55 | LC | 24 |
|  | Selar crumenophthalmus | Carangidae | 3.7 | 3 | 78 | LC | 39 |
|  | Seriola rivoliana | Carangidae | 4.5 | 5.3 | 81 | LC | 76 |
|  | Seriolina nigrofasciata | Carangidae | 3.8 | 2.4 | 73 | LC | 38 |
|  | Trachinotus africanus | Carangidae | 4.4 | 3 |  | LC | 47 |
|  | Trachinotus baillonii | Carangidae | 4.5 | 2.1 | 64 | LC | 36 |
|  | Trachinotus botla | Carangidae |  | 2.5 |  | LC | 40 |
|  | Ulua mentalis | Carangidae |  | 3.2 |  | LC | 51 |
|  | Uraspis secunda | Carangidae |  | 1.8 | 76 | LC | 33 |
|  | Aprion virescens | Lutjanidae | 4.3 | 4.3 | 66 | LC | 61 |
|  | Apsilus fuscus | Lutjanidae | 4 | 3.2 | 46 | LC | 50 |
|  | Etelis carbunculus | Lutjanidae | 4.5 | 1.8 | 59 | LC | 44 |
|  | Etelis coruscans | Lutjanidae | 4.4 | 2.4 | 70 | LC | 45 |
|  | Lutjanus bengalensis | Lutjanidae | 3.8 | 1.6 |  | LC | 27 |
|  | Lutjanus bohar | Lutjanidae | 4.3 | 7.2 | 64 | LC | 69 |
|  | Lutjanus ehrenbergii | Lutjanidae | 0 | 0.8 | 48 | LC | 19 |
|  | Lutjanus fulviflamma | Lutjanidae | 3.8 | 2.4 | 66 | LC | 34 |
|  | Lutjanus fulvus | Lutjanidae | 3.6 | 1.7 | 48 | LC | 29 |
|  | Lutjanus gibbus | Lutjanidae | 4.1 | 2.2 | 68 | LC | 35 |
|  | Lutjanus johnii | Lutjanidae | 4.2 | 4 | 61 | LC | 54 |
|  | Lutjanus kasmira | Lutjanidae | 3.9 | 3.3 | 70 | LC | 40 |
|  | Lutjanus lunulatus | Lutjanidae | 4 | 2 | 45 | LC | 33 |
|  | Lutjanus lutjanus | Lutjanidae | 4 | 1.4 | 58 | LC | 25 |
|  | Lutjanus monostigma | Lutjanidae | 4.3 | 3 | 58 | LC | 40 |
|  | Lutjanus rivulatus | Lutjanidae | 4.1 | 2.9 | 66 | LC | 42 |
|  | Lutjanus russellii | Lutjanidae | 4.1 | 2.3 | 69 | LC | 36 |
|  | Lutjanus sanguineus | Lutjanidae | 4.5 | 4.1 | 61 | LC | 56 |
|  | Lutjanus sebae | Lutjanidae | 4.1 | 3.9 | 70 | LC | 59 |
|  | Lutjanus vitta | Lutjanidae | 4 | 3.1 | 61 | LC | 38 |
|  | Macolor niger | Lutjanidae | 4 | 3.2 | 66 | LC | 47 |
|  | Paracaesio xanthura | Lutjanidae | 3.4 | 2.3 | 70 | LC | 36 |
|  | Pinjalo pinjalo | Lutjanidae | 3.4 | 3.3 | 58 | LC | 49 |
|  | Pristipomoides filamentosus | Lutjanidae | 4.2 | 2.7 | 61 | LC | 43 |
|  | Pristipomoides multidens | Lutjanidae | 3.8 | 2.9 | 68 | LC | 43 |
|  | Pristipomoides sieboldii | Lutjanidae | 3.7 | 1.9 | 67 | LC | 36 |
|  | Pristipomoides typus | Lutjanidae | 4.2 | 2.6 | 44 | LC | 39 |
|  | Pristipomoides zonatus | Lutjanidae | 4 | 2.9 | 64 | LC | 42 |
|  | Aphareus furca | Lutjanidae | 4.1 | 2 | 66 | LC | 36 |
|  | Aphareus rutilans | Lutjanidae | 4.1 | 3.7 | 70 | LC | 58 |
|  | Aprion virescens | Lutjanidae | 4.3 | 4.3 | 66 | LC | 61 |
| Large Pelagic Fish | Acanthocybium solandri | Scombridae | 4.3 | 1.7 | 107 | LC | 46 |
|  | Auxis rochei | Scombridae | 4.5 | 1.1 | 112 | LC | 27 |
|  | Auxis thazard | Scombridae | 4.5 | 0.9 | 112 | LC | 28 |
|  | Euthynnus affinis | Scombridae | 4.4 | 1.1 | 73 | LC | 36 |
|  | Gymnosarda unicolor | Scombridae | 3.2 | 4.6 | 61 | LC | 73 |
|  | Katsuwonus pelamis | Scombridae | 4.2 | 1.3 | 110 | LC | 38 |
|  | Rastrelliger kanagurta | Scombridae | 4.5 | 0.7 | 74 | DD | 21 |
|  | Sarda orientalis | Scombridae | 4.2 | 2.3 | 84 | LC | 50 |
|  | Scomberomorus commerson | Scombridae | 4.3 | 2.1 | 80 | NT | 52 |
|  | Scomberomorus plurilineatus | Scombridae | 4.4 | 1.1 | 36 | DD | 39 |
|  | Thunnus alalunga | Scombridae | 4.5 | 2.7 | 110 | NT | 58 |
|  | Thunnus albacares | Scombridae |  | 1.8 | 107 | NT | 51 |
|  | Thunnus obesus | Scombridae |  | 2.3 | 100 | VU | 56 |
|  | Rachycentron canadum | Rachycentridae | 4 | 1.4 | 84 | LC | 44 |
|  | Xiphias gladius | Xiiphidae | 4.5 | 4.5 | 119 | LC | 72 |
| Demersals (skates and rays) | Okamejei heemstrai | Rajidae |  | 2.5 |  | LC | 44 |
|  | Aetobatus narinari | Aetobatidae | 4.2 | 5.2 |  | LC | 75 |
|  | Torpedo sinuspersici | Torpedinidae | 4.5 | 9.8 | 61 | DD | 76 |
|  | Himantura uarnak | Dasyatidae | 3.6 |  | 75 | VU | 90 |
|  | Maculabatis ambigua | Dasyatidae | 3.7 |  |  | NT | 66 |
|  | Mobula kuhlii | Mobulidae | 3.4 |  |  | EN | 73 |
|  | Pateobatis jenkinsii | Dasyatidae | 0 |  | 58 | LC | 78 |
|  | Rhinoptera javanica | Rhinopteridae | 3.3 |  | 45 | VU | 90 |
|  | Rhinoptera jayakari | Rhinopteridae | 3.6 |  |  | NE/DD | 66 |
|  | Taeniura lymma | Dasyatidae | 3.6 |  | 62 | LC | 90 |
|  | Taeniurops meyeni | Dasyatidae | 4.2 |  | 77 | VU | 77 |
| Cephalopods | Octopus cyanea | Octopodidae |  |  | 69 |  | 72 |
|  | Octopus vulgaris | Octopodidae | 3.35 | 1.2 | 95 |  | 78 |
|  | Sepia latimanus | Sepiidae |  | 1.5 | 66 |  | 40 |
|  | Loligo forbesi | Loliginidae | 4.15 |  | 89 | LC | 56 |
|  | Loligo duvaucelii | Loliginidae |  | 1 | 45 | DD | 14 |

| Table S7: Life history trait values (FishBase) averaged from all individual species for each fish functional group used in the study. | | | | | |
| --- | --- | --- | --- | --- | --- |
| **Functional Group** | **Family** | **Trophic level** | **Age at first maturity** | **Latitudinal Range** | **Vulnerability score** |
| Small Pelagic Fish | Clupeidae | 2.95 | 0.517 | 54.00 | 13.33 |
|  | Engraulidae | 3.267 | 0.922 | 62.33 | 16.22 |
| Coral Reef Fish | Siganidae | 2.225 | 1.675 | 50.25 | 27.75 |
|  | Lethrinidae | 3.815 | 2.625 | 58.80 | 38.75 |
|  | Carangidae | 3.968 | 2.219 | 70.64 | 39.30 |
|  | Lutjanidae | 3.91 | 2.884 | 61.80 | 42.77 |
| Large Pelagic | Scombridae | 4.273 | 1.823 | 89.69 | 44.23 |
|  | Rachycentridae | 4 | 1.4 | 84.00 | 44.00 |
|  | Xiiphidae | 4.5 | 4.5 | 119.00 | 72.00 |
| Demersals | All families | 3.41 | 5.833 | 63.00 | 75.00 |
| Cephalopods | Octopus | 3.35 | 1.2 | 82.00 | 75.00 |
|  | Squid | 4.15 | 1.25 | 66.67 | 36.67 |

**References:**

Froese, R. and D. Pauly. Editors. 2023.FishBase. World Wide Web electronic publication.
www.fishbase.org, (02/2023)

Heck, N., Agostini, V., Reguero, B. G., Pfiegner, K., Mucke, P., Kirch, L., & Beck, M. W. (2020). Fisheries at Risk: Vulnerability of Fisheries to Climate Change. *The Nature Conservancy, Techincal Report*.

*WHO* 2020. Life expectancy at birth,World Health Organization. Available at: https://www.who.int/data/gho/data/indicators/indicator-details/GHO/life-expectancy-at-birth-(years).
